# The emergence of hyperendemic dengue in Bangladesh: An ecological study of structural breaks and transmission regime shifts, 2008–2025

**DOI:** 10.1371/journal.pone.0343246

**Published:** 2026-02-20

**Authors:** Pratyay Hasan, Tazdin Delwar Khan, Mohd. Arifuzzaman, Md. Saiful Islam, Mohammad Emdadul Haque

**Affiliations:** 1 Emergency Department, Dhaka Dental College and Hospital, Dhaka, Bangladesh; 2 Department of Cardiac Anesthesiology, Ibrahim Cardiac Hospital and Research Institute, Dhaka, Bangladesh; 3 Department of Public Health, North Western University, Khulna, Bangladesh; 4 OSD, Directorate General of Health Services (DGHS) and Adjunct Faculty, Department of Public Health, Bangladesh Open University, Gazipur, Bangladesh; 5 Department of Maxillofacial Surgery, Bangabandhu Sheikh Mujib Medical University, Dhaka, Bangladesh; GMC Kollam: Government Medical College Kollam, INDIA

## Abstract

**Background:**

Dengue fever in Bangladesh has escalated from sporadic outbreaks to a persistent, nationwide health crisis. Traditional epidemiological analyses often assume a constant transmission regime, potentially overlooking fundamental shifts driven by viral, environmental, or societal factors. This population-level ecological time-series observational study aimed to identify and characterize significant structural breaks in the time series of dengue admissions in Bangladesh to define distinct epidemiological phases.

**Methods:**

Monthly dengue hospital admission data (January 2008**─**October 2025) were obtained from the Institute of Epidemiology, Disease Control and Research (IEDCR) and Directorate General of Health Services (DGHS) public archives. Analyses included STL seasonal-trend decomposition, the Zivot-Andrews unit root test (primary break detection), multi-algorithm breakpoint detection (PELT, Binary Segmentation, Window-based), K-means clustering (optimal at 3 clusters, silhouette score 0.867), and Markov regime-switching models.

**Findings:**

Ten structural breaks were identified through a consensus ranking approach. The most prominent break occurred in May 2021 (consensus score = 3). The Markov regime-switching model delineated three distinct transmission regimes: 1) a Low Baseline Regime (2008**─**2023) with a mean of 47 monthly cases (95% CI: 36**─**57); 2) an Intermediate Regime (2008**─**2025) with a mean of 1,288 monthly cases (95% CI: 927**─**1,648); and 3) a Hyperendemic Regime (2019**─**2025) with a mean of 26,127 monthly cases (95% CI: 17,207─35,048), representing a 556-fold increase over the low baseline. Seasonality strength was moderate (0.335), but the peak-to-trough seasonal ratio approached 180, indicating pronounced annual epidemic cycles superimposed on the substantially elevated baseline.

**Interpretation:**

Bangladesh has experienced an established regime shift to sustained hyperendemic dengue transmission (persistent as of October 2025) necessitating a fundamental shift from outbreak-response to sustained, year-round control strategies. It is most likely influenced by viral, environmental, and societal factors including documented serotype redistribution. Public health strategies must transition from outbreak-response to sustained high-transmission management, including year-round vector control with pre-monsoon intensification.

## Introduction

Dengue fever has been a continued public health problem in Bangladesh, evolving from a series of localized outbreaks into a persistent, nationwide crisis [1]. The year 2023 marked the highest recorded burden, with a staggering 73,023 admissions reported in September alone [2] and this elevated burden has persisted through 2024–2025, confirming a fundamental shift in transmission dynamics. This study utilizes complete surveillance data through October 2025, providing the most current assessment of Bangladesh’s dengue trajectory and confirming the sustained nature of recent epidemiological shifts.

This is a population-level ecological time-series observational study relying on aggregated national monthly dengue hospital admission data from the Directorate General of Health Services (DGHS) and Institute of Epidemiology, Disease Control and Research (IEDCR) passive surveillance system. This system captures reported cases from all hospitals under DGHS (both public and private), with likely underreporting of milder community cases. The study population is the national monthly count of hospital-admitted dengue cases.

Conventional analyses of disease trends often rely on the assumption of a single, stable state or a smooth, linear progression. However, the dynamics of complex infectious diseases like dengue are influenced by a confluence of factors including, viral evolution (e.g., serotype redistribution) [3], climatic conditions [4], rapid urbanization [5], and vector adaptability—all of which can cause sudden, fundamental, and sustained changes in transmission dynamics. These changes, known as structural breaks or regime shifts, render time-series models and public health strategies based on prior states obsolete.

The Zivot-Andrews unit root test with endogenous structural break detection is superior to standard trend-stationary models in this context, as it avoids assuming a priori break dates and better distinguishes true non-stationary regime changes from inherent seasonality or gradual trends that might otherwise be misattributed [6,7]. This approach is complemented by multi-algorithm consensus breakpoint detection, K-means clustering, and Markov regime-switching models to provide robust delineation of epidemiological phases.

The major mid-2023 structural break identified in this analysis temporally aligns with well-documented literature on the re-emergence of DENV-2 as the predominant circulating serotype in Bangladesh during 2023 (reported at approximately 74% in key molecular studies, following DENV-3 dominance from 2019 to 2022) [8]. This serotype shift is associated with increased transmission intensity and clinical severity, potentially due to mechanisms such as antibody-dependent enhancement in populations with prior exposure to heterologous serotypes [8].

While the increasing case numbers in Bangladesh are evident, the nature of this escalation, whether gradual or punctuated by distinct phases, has not been formally characterized through rigorous statistical testing for structural breaks. Understanding this trajectory is critical for accurate risk assessment and proactive policy formulation.

Bangladesh serves as a critical case study for hyperendemic dengue evolution in South Asia. Identifying these regimes enables the shift from reactive “outbreak-response” policies to proactive “adaptive” strategies that address a sustained high-transmission baseline (e.g., year-round vector control and permanent clinical capacity). This methodology can also be applied to other hyperendemic regions to detect epidemiological tipping points. The findings must be viewed in the context of the global dengue surge, which reached a record high of approximately 14 million cases in 2024 (with over 9,000 deaths reported), driven by similar drivers of climate variability, urbanization, and vector expansion [9,10].

This study aims to formally identify and characterize the significant structural breaks in the monthly dengue admission time series for Bangladesh from 2008 to 2025. Our objective is to delineate distinct epidemiological phases defined by these breaks and to interpret their public health implications, providing a data-driven narrative of the disease’s evolution.

## Methods

### Study design and data source

This study constitutes a retrospective time-series analysis of secondary, national-level data. The primary outcome was monthly dengue admission data spanning from January 2008 to October 2025. Data were collected from the publicly available archives and situation reports of the Institute of Epidemiology Disease Control and Research (IEDCR) and the Directorate General of Health Services (DGHS) [2,11]. This passive surveillance system primarily captures reported cases from all hospitals under DGHS (both public and private), with likely underreporting of milder community cases. Data included complete surveillance records through October 2025, representing actual reported cases rather than projections or forecasts. No monthly values were missing in the compiled national aggregates, so no imputation was required. This complete dataset enables confirmation of sustained transmission patterns and regime stability.

The reporting of this study follows the STROBE guidelines for observational studies.

### Analytical framework: STL decomposition

To isolate and quantify the underlying patterns in the monthly dengue case data, we employed the STL (Seasonal-Trend decomposition using LOESS) method. STL is a robust and versatile filtering procedure for decomposing a time series into three additive components:


$$Y_{t}=T_{t}+S_{t}+R_{t}$$

Where:

- *Y*_*t*_ is the observed dengue case count at time *t*.
- *T*_*t*_ is the Trend-cycle component, representing long-term progression (e.g., multi-year increases or decreases).
- *S*_*t*_ is the Seasonal component, representing regular, fixed-period fluctuations (e.g., annual cycles).
- *R*_*t*_ is the Remainder (or residual) component, containing the irregular noise or randomness not explained by the trend or seasonality.

An additive model was selected because visual inspection and component strength metrics showed relatively stable seasonal amplitude compared with the explosive trend component. The STL algorithm employs an iterative, two-loop process using LOESS (Locally Estimated Scatterplot Smoothing) regression and robustness weights to ensure the decomposition is not skewed by outliers. Implementation parameters in Python’s statsmodels library were: period = 12, seasonal = 13, and robust = True.

To provide objective metrics, we calculated the strength of the trend (*F*_*T*_) and seasonality (*F*_*S*_) using established formulae:

**Trend strength:**


$$F_{T}=max(\text{0},\text{1}-\frac{\text{Var}(R_{t})}{\text{Var}(T_{t}+R_{t})})$$

**Seasonality strength:**


$$F_{S}=max(\text{0},\text{1}-\frac{\text{Var}(R_{t})}{\text{Var}(S_{t}+R_{t})})$$

Where, Var(⋅) denotes the sample variance. Values close to 1 indicate the component dominates the remainder, signifying a strong pattern relative to the noise.

### Statistical analysis: Structural break identification and regime delineation

1 **Structural Break Detection**

**Primary Break Detection:** To formally test for fundamental shifts, we employed the Zivot-Andrews test [7], a robust procedure that endogenously identifies a single unknown breakpoint, thus avoiding the need for *a priori* specification of break dates. The test is based on an augmented regression model that incorporates dummy variables to capture a break in both the level (intercept) and the trend of the series:


$$\Delta y_{t}=\alpha +\beta t+\gamma y_{t-\text{1}}+\theta {\text{DU}}_{t}(\lambda )+\omega {\text{DT}}_{t}(\lambda )+\sum_{j=\text{1}}^{k}d_{j}\Delta y_{t-j}+\varepsilon_{t}$$

Here:

- *t* is a linear time trend.
- *DU*_*t*_ (λ) is an intercept break dummy.
- *DT*_*t*_ (λ) is a trend break dummy.

The null hypothesis (H_0_: α = 0) posits a unit root without a break, while the alternative (H_1_: α < 0) indicates a trend-stationary process with a break at time *T*_*B*_. The break date λ is selected to minimize the one-sided t-statistic for α, identifying the most likely point of structural change. Statistically significant breakpoints (p < 0.05) were subsequently interpreted as the start of new epidemiological phases.

**Multi-Algorithm Consensus Approach:** To robustly identify multiple regime shifts, we implemented a consensus-based multiple breakpoint detection framework combining three state-of-the-art change-point algorithms after the primary Zivot-Andrews test:

- **PELT** (Pruned Exact Linear Time) – optimal for known number of changes [12]
- **Binary Segmentation** – fast heuristic for multiple breaks [13]
- **Window-based segmentation** – sliding window comparison [14]

All algorithms used radial basis function (RBF) cost functions with min_size = 12 months. Breakpoints were ranked by consensus score (frequency across methods) and magnitude of mean change (pre- vs post-break). Breaks detected by ≥2 algorithms or showing large mean ratio changes were prioritized. The top-ranked breaks defined candidate epidemiological transition points.

2 **Transmission Regime Identification**

Two complementary approaches were used to delineate distinct epidemiological regimes:

1. **Unsupervised clustering of transmission features** (K-means, k = 2–5) on log-transformed case counts, 12-month rolling mean, coefficient of variation, and outbreak intensity (z-score > 2). Optimal cluster number was determined via silhouette score.
2. **Markov-switching regression model** [15] with k = 3 regimes, allowing switching mean and variance:

$$y_{t}=\mu_{s_{t}}+\varepsilon_{t},\varepsilon_{t}|s_{t}\sim N(\text{0},\sigma_{s_{t}}^{\text{2}}),s_{t}\in \{\text{1},\text{2},\text{3}\}$$

$y_{t}$ is the unobserved regime at time *t*, governed by a first-order Markov chain. Smoothed regime probabilities were used to assign dominant regimes and detect transition points.

Previously, unsupervised clustering (e.g., K-means) has been successfully applied to epidemic time-series to classify outbreak patterns (e.g., “-outbreak types”- during COVID-19) [16]. Separately, Markov-switching (regime-switching) models have long been used to detect epidemic vs non-epidemic phases in surveillance data, allowing for switching mean and variance across latent states (e.g., influenza surveillance) [17].

The Markov regime-switching model served as the primary method for defining final epidemiological regimes, as it accounts for temporal dependence and provides smoothed probabilistic state assignments. K-means clustering and structural break analysis provided complementary validation. This multi-method synthesis allowed ten structural breaks to be identified and coalesced into three main epidemiological regimes.

3 **Unit Root and Stationarity Testing with Structural Breaks**

Series non-stationarity was assessed using:

- Zivot–Andrews test (Model C: break in intercept and trend) [7]
- Augmented Dickey–Fuller (ADF) [18]
- Phillips–Perron (PP) [19]
- DF-GLS tests [20]

A significant Zivot–Andrews test (p < 0.05) confirmed the presence of trend-stationarity with endogenous structural break(s).

4 **Epidemiological Metrics and Outbreak Definition**

Key metrics included:

- Coefficient of variation (CV)
- Annual growth rate (linear trend coefficient × 12/ mean × 100)
- Outbreak frequency: proportion of months exceeding 2σ above 12-month centered rolling mean
- Seasonal ratio: peak vs trough monthly mean

### Software and reproducibility

All analyses were implemented in Python 3.13 using:

- Pandas, numpy, matplotlib, seaborn
- Statsmodels (STL, Zivot–Andrews, MarkovRegression) [21].
- Ruptures (PELT, BinSeg, Window)
- Scikit-learn (K-means, silhouette score)
- Arch (Phillips–Perron, DF-GLS)

### Ethics statement

This study analyzed exclusively aggregated, anonymized, and publicly available data from official government portals. No individual-level data was accessed. Following international guidelines (e.g., CIOMS) for epidemiological research using public surveillance data, this study was considered exempt from ethical board approval. The research was conducted with rigorous adherence to scientific integrity and the principles of the Declaration of Helsinki.

### Conceptual framework

Structural breaks are points in time when the underlying parameters of dengue transmission shift sustained, usually due to events such as viral change, climatic variation, or major societal disruptions [22]. Transmission regimes are the sustained periods that follow these breakpoints and are defined by stable statistical properties such as mean incidence and seasonal pattern [23,24]. Breaks mark the transition, and regimes describe the new operating state of the system. These shifts in Bangladesh likely reflect combined effects of viral evolution, climate change, rapid urbanization, population mobility, and external shocks such as the COVID lockdown [25]. Distinguishing between breaks and regimes is essential for interpretation of dengue trends. Breaks provide early warning of fundamental change, while regimes offer the basis for long term planning and program design.

## Results

The temporal trajectory of dengue admissions in Bangladesh from 2008 to 2025 is characterised by distinct epidemiological regimes, marked by significant structural breaks. The comprehensive multi-method analysis confirmed non-stationarity and identified ten structural breaks, with a consensus around key transitions.

### Seasonality and trend components

STL decomposition showed a highly dominant trend component (high trend strength) and moderate seasonality strength (0.335), indicating that long-term trend variability predominates over regular seasonal fluctuations in explaining overall series variance.

Nevertheless, the peak-to-trough seasonal ratio reached approximately 180, highlighting extremely intense amplification during annual monsoon epidemic cycles atop the elevated post-2023 baseline. The moderate seasonality strength value indicates the explosive trend dominates total variance, while the high seasonal ratio confirms that within each year, the monsoon-driven peak is massively amplified relative to the new, elevated baseline.

Overall series metrics (January 2008–October 2025): total cases 711,618, mean monthly ≈3,325, SD ≈ 10,727, CV ≈ 3.23, maximum monthly 73,023, minimum monthly 0. Linear trend coefficient ≈63.9 cases/month, estimated annual growth ≈23.1%, doubling time ≈3.0 years (see Supplementary epidemiological metrics).

### Structural breaks and epidemiological phases

The Zivot-Andrews test confirmed a significant unit root with a structural break (Test Statistic: −8.277, p < 0.001). The multi-algorithm consensus approach identified ten structural breaks, with the most statistically significant breaks occurring in 2021−05, 2020−02, 2015−07, and 2011−05.

### Structural breaks

Structural Breaks The consensus-based multi-algorithm approach (PELT, Binary Segmentation, Window-based segmentation) identified ten significant structural breaks in the monthly dengue admission time series (Supplementary Table S1 File). Breaks were ranked by consensus score (number of algorithms detecting the date) and magnitude of mean change. The highest-consensus break was in May 2021 (consensus score 3, detected by all three algorithms), with pre-break mean ≈819 cases/month increasing to post-break mean ≈10,750 (ratio ≈13.1, magnitude change ≈9,931).

**Other prominent breaks included:**

- July 2015 (score 2, ratio ≈82.6, magnitude ≈5,620)
- February 2020 (score 2, ratio ≈9.4, magnitude ≈7,536)
- June 2023 (score 1, ratio ≈14.0, magnitude ≈15,660)
- September 2024 (score 1, ratio ≈4.3, magnitude ≈8,946)

The full list of ten breaks, including dates, consensus scores, detecting methods, pre- and post-break means, ratios, and magnitude changes, is provided in Supplementary Table S1 File. K-means clustering applied to regime features produced an optimal solution of three clusters (silhouette score 0.867, the highest among 2–5 clusters evaluated; see Supplementary Table S2 File). This clustering solution was supported and refined by Markov regime-switching models.

The ten structural breaks were synthesized into three distinct epidemiological regimes (Supplementary Table S4 File) (Table 1):

**Table 1:** Identified transmission regimes in dengue admissions (2008-2025) based on markov regime-switching model.

Regime	Period	Expected persistence of the regime* (months)	Interpretation	Mean Monthly Cases (95% CI)	Key Characteristics
0	2008–01–01–2023–04–01	**129**	**Low Baseline Regime**	**47 (36–57)**	Very low, stable transmission; sporadic outbreaks; high variability relative to mean (CV = 1.31)
1	2008–08–01–2025–06–01	**61**	**Intermediate Regime**	**1,288 (927–1,648)**	Moderate, fluctuating transmission; transitional phase with elevated baseline; increased volatility (CV = 1.09)
2	2019–07–01–2025–10–01	**24**	**Hyperendemic Regime**	**26,127 (17,207–35,048)**	Sustained high transmission; extreme seasonal peaks; represents a **556-fold increase** over the low baseline regime; dramatic elevation in expected outbreak scale

* The listed durations represent the estimated average (expected) persistence of each regime (i.e., the mean time spent in a regime before switching, calculated as 1/(1 – p_ii_) where p_ii_ is the regime’s self-transition probability), not the total calendar span of observations assigned to that regime [15,26]. The much longer calendar spans reflect cumulative assignments across the series, while the shorter listed values indicate typical regime dwell times under the model’s dynamics [15,26,27].

The visual progression of these regimes, with their corresponding changes in mean and variance, is presented in. The most critical shift occurred in mid-2019, initiating the Hyperendemic Regime, which dramatically and sustainedly elevated the expected scale of outbreaks, as evidenced by the historic peak of 73,023 monthly admissions in September 2023. The Hyperendemic Regime has sustained a mean of 26,127 monthly cases—a 556-fold increase over the Low Baseline Regime—and has persisted through October 2025 (although expected persistence being 24 months). This aligns with spatial studies confirming nationwide dissemination during this period (Hossain et al., 2023). The Markov Regime-Switching and clustering models confirm that, after the extreme 2023–2024 peak, Bangladesh has remained locked in a dramatically elevated transmission state through October 2025.

**Fig 1:**
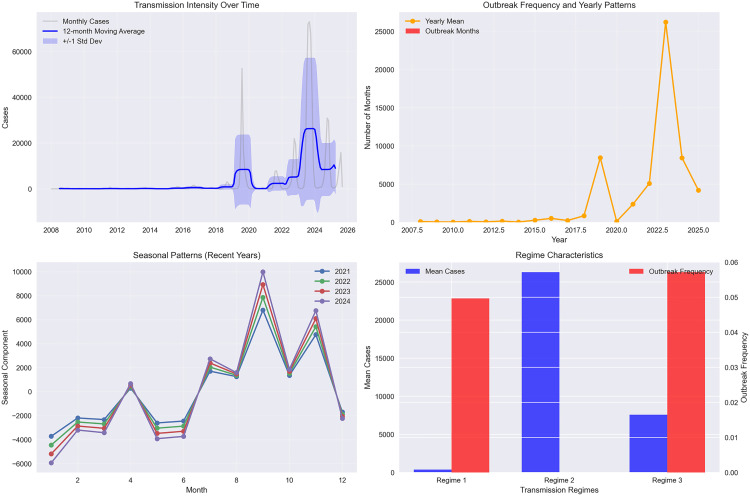
Epidemiological metrics and seasonality analysis in Bangladesh (2008-2025). Fig 1 integrates four complementary panels that together demonstrate a profound structural shift in dengue transmission in Bangladesh. The top-left panel shows that monthly admitted cases stayed low and stable until 2018, rose sharply with the 2019 escalation, peaked explosively in 2023, and then settled into a new but still highly elevated baseline. The top-right panel reveals that yearly outbreak patterns have shifted from rare, isolated events before 2019 to near-continuous outbreak conditions thereafter, with 2023 exhibiting unprecedented sustained transmission. The bottom-left panel shows that seasonality remains strongly monsoon-driven and highly consistent, though now sitting atop a far higher baseline. Finally, the bottom-right panel uses clustering to identify three clear transmission regimes (from unsupervised k means clustering)—low-endemic, transitional escalation, and hyperendemic—indicating that the system no longer returns to its pre-2019 state. Taken together, these panels provide compelling visual evidence of a lasting structural shift toward sustained hyperendemic dengue transmission.

**Fig 2:**
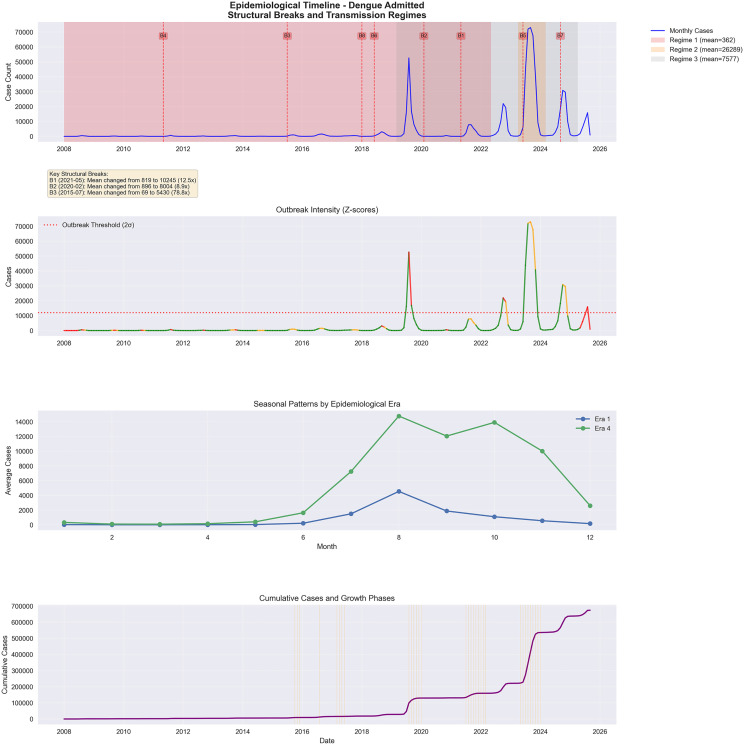
Epidemiological timeline of monthly dengue admissions in Bangladesh (2008-2025, October). This figure presents a unified epidemiological timeline across four panels, clearly demonstrating the structural transformation of dengue transmission in Bangladesh. The top panel overlays monthly cases with data-driven regime classifications (from unsupervised k means clustering) and detected breakpoints, showing three major phases: a long low-endemic era, a sharp escalation beginning around 2019–2021, and a sustained hyperendemic period through 2024–2025, all marked by abrupt, algorithm-identified shifts. The second panel displays outbreak intensity using Z-scores, revealing that months exceeding the + 2σ threshold were almost absent before 2019 but became persistent and extreme from 2022 onward, confirming that outbreaks transitioned from rare anomalies to the default state. The third panel compares seasonal patterns across eras, showing that the monsoon-driven peak remains intact but is now magnified by an order of magnitude in the hyperendemic era. Finally, the bottom panel charts cumulative dengue cases, which remained nearly flat for a decade before accelerating sharply in alignment with the identified structural breaks, forming a clear “hockey-stick” trajectory. Together, these panels provide strong, convergent evidence of a sustained shift to high, structurally altered dengue transmission.

**Fig 3:**
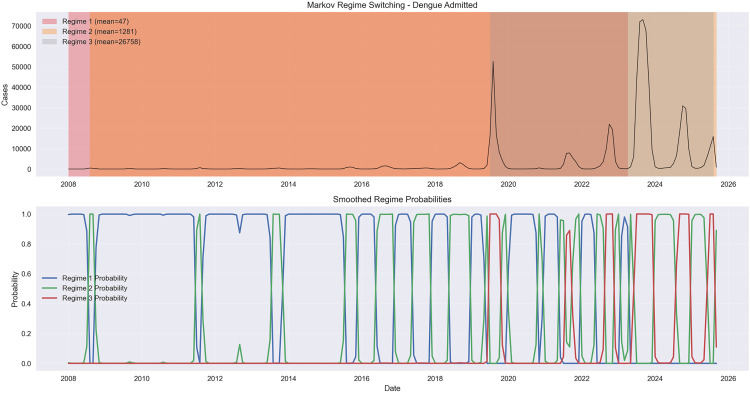
Markov regime switching analysis. This figure uses a Markov regime-switching framework to identify dengue transmission phases, and both panels show a clear, escalating shift toward hyperendemicity. In the top panel, the model classifies monthly dengue cases into three distinct regimes—an extremely low baseline through mid-2019, a sharply elevated intermediate phase through ~2022, and a dramatic hyperendemic regime thereafter—mirroring the same structural jumps detected by all other analytical methods. The bottom panel, which plots smoothed regime probabilities, reveals how these transitions unfolded: the probability of the old low-endemic regime stayed near 1.0 until 2018, collapsed abruptly around 2019 as the intermediate phase took over, and then gave way to the hyperendemic regime, whose probability rises to nearly 1.0 from 2022 onward and remains dominant even during the 2023 peak. Together, the two panels show that the transition was not gradual drift but a sequence of decisive, model-verified shifts culminating in a stable hyperendemic state.

The progression through these regimes shows an alarming acceleration: the Low Baseline Regime persisted for approximately 15 years, the Intermediate Regime overlapped throughout much of the study period, while the Hyperendemic Regime has established a new, sustained baseline.

## Discussion

The progression of dengue in Bangladesh reflects a sequence of discrete and lasting regime shifts rather than a gradual linear rise, a pattern made clear through our comprehensive structural break and regime-switching analysis. We identified ten structural breaks and three distinct transmission regimes defined by the Markov model, a finding that significantly refines the earlier understanding of a single hyperendemic shift [1,28]. These findings overturn the assumption of a single stable transmission state that is often embedded in conventional forecasting approaches. When contrasted with forecasting models [28–30] the structural breaks show that any forecasting method that assumes a constant underlying regime will be inherently limited and must eventually incorporate phase-specific adjustment and regime shift detection.

The most pivotal transition is the hyperendemic shift of mid-2019, which has established a sustained, elevated baseline for dengue outbreaks. The Hyperendemic Regime reached a mean of 26,127 monthly cases (95% CI: 17,207–35,048)—a 556-fold increase over the Low Baseline Regime—and has persisted through October 2025. Furthermore, the identification of a structural break in September 2024 signals a concerning development: intensification within the hyperendemic phase itself. This break, with a 4.3-fold increase in mean monthly cases (from 2,740–11,686), suggests that the new high-transmission state is not stable but may be experiencing further escalation, demanding even more aggressive public health measures.

These findings align with epidemiological evidence describing the nationwide spread of dengue into all 64 districts in 2023 [1]. Virological observations further suggest that the re-emergence and subsequent dominance of DENV-2 during this period contributed to this dramatic elevation in transmission intensity and clinical severity [8]. The temporal alignment of the June 2023 structural break with serotype redistribution underscores the role of viral evolution in driving regime shifts.

The extraordinary seasonal ratio (≈180) underscores the critical importance of pre-monsoon and early monsoon vector control interventions, as this predictable seasonality provides a clear timeframe for targeting public health resources most effectively. However, the moderate seasonality strength (0.335) relative to the dominant trend indicates that while annual peaks are extreme, the underlying elevated baseline is the primary driver of overall disease burden. This implies that interventions must now be year-round, with pre-monsoon intensification (March–May) critical to mitigating the explosive August peak.

The earlier breaks that preceded the 2019 shift correspond closely to national epidemiological transitions reported in the literature. The phases associated with endemic establishment after 2008 and intensification after 2011 match the findings of Mutsuddy et al. (2019), who noted that the traditional July to October peak began expanding into the pre-monsoon months from 2014 onward [31,32]. Their work highlighted that pre-monsoon dengue incidence during 2015–2017 was more than seven times higher than in the earlier fourteen-year period, attributing this shift to climatic variability, ecological imbalance, rapid urbanization, and strong correlations with humidity. Earlier environmental modelling work supports the contribution of temperature, rainfall, and humidity to these transitions [4]. The multiple structural breaks add statistical confirmation by aligning these epidemiological changes with clear, successive shifts in the underlying data-generating process, culminating in the current hyperendemic regime. The Markov model identified an ‘Intermediate Regime’ with high volatility that overlapped temporally with both the Low and Hyperendemic regimes. This likely represents a prolonged transitional period of increasing instability in the transmission system, rather than a stable state with distinct temporal boundaries.

The most significant finding of this analysis is the confirmation that Bangladesh’s dengue transmission has undergone a fundamental and sustained regime shift. Unlike previous outbreak years that returned to lower baselines, the 2019–2025 period demonstrates sustained hyperendemic transmission. This represents a ‘new normal’ that requires fundamental rethinking of public health responses. The Hyperendemic Regime has sustained a mean monthly case load of 26,127, which represents a 556-fold increase over the low baseline phase. This markedly elevated transmission has now persisted so long, covering multiple full seasonal cycles and indicating that it is not a temporary anomaly.

### Addressing surveillance bias

While improvements in diagnostic capacity and reporting over the 18-year period may contribute to observed trends, the scale of the surge—particularly the 556-fold increase from the low baseline to the hyperendemic regime—argues strongly against artifact alone. Parallel evidence from hospital burden, mortality trends, and media reports during the 2023–2024 peak supports the reality of a true epidemiological escalation beyond improved detection [33]. Furthermore, structural breaks represent sudden changes in the data-generating process; gradual improvements in surveillance would be unlikely to produce such sharp, statistically identifiable discontinuities.

The implications for public health strategy are substantial. Dengue transmission in Bangladesh now operates under a new hyperendemic regime with a dramatically elevated baseline (mean ≈ 26,127 monthly cases). Preparedness strategies must therefore assume this consistently higher and more volatile baseline, rather than relying on assumptions drawn from earlier, lower-intensity phases.

### Public health implications

The identification of these structural breaks and the confirmed sustained hyperendemic regime has profound implications for public health practice:

- Forecasting and Surveillance: Models that assume a single, stable state are inherently unreliable. Future forecasting efforts must be regime-specific and incorporate regime-shift detection algorithms to provide accurate early warnings. The historical data from the pre-2023 regimes is no longer a valid benchmark for predicting outbreak magnitude in the current hyperendemic regime. The sustained transmission through October 2025 provides the new baseline for all future projections. The detection of a break within the hyperendemic phase in September 2024 underscores the need for continuous, real-time surveillance to detect further escalations.
- Preparedness and Resource Allocation: The Hyperendemic Regime (2023–2025) provides the new baseline for preparedness. Health system capacity, including hospital beds, ICU facilities, and medical supplies, must be permanently scaled to the reality of sustained, high-level transmission with a new baseline of approximately 26,127 mean monthly cases. The confirmation that this elevated state has persisted for more than 2 years means budgetary planning must reflect this sustained increase in case load, not just seasonal surge capacity.
- Intervention Strategy: Vector control and public health messaging strategies must be fundamentally re-evaluated for efficacy in a sustained high-transmission regime. Approaches designed for a lower-intensity endemic state are completely insufficient and need to be replaced with more aggressive, sustained, and potentially novel interventions tailored to this new reality. The intense seasonality (seasonal ratio ≈ 180) suggests that targeted, high-intensity vector control in the months leading up to the August peak could yield significant reductions in disease burden, but must be implemented at a scale appropriate for the new hyperendemic baseline. The 2024 break indicates that even current control measures may be inadequate to contain the intensifying transmission. Interventions must now be year-round, with pre-monsoon intensification (March–May) critical to mitigating the explosive August peak.

### Strengths and limitations

As an ecological study using aggregated national surveillance data, causal inference on drivers (e.g., serotype changes, climate, urbanization) is limited to temporal alignment with published literature. The univariate time-series approach does not directly incorporate covariate data. Passive surveillance likely results in substantial underreporting (especially of mild or non-hospitalized cases), and diagnostic/reporting improvements may contribute to apparent trends — though the scale of the surge argues strongly against artifact alone. Future research integrating this breakpoint framework with hierarchical models incorporating spatially-explicit climate data, serotype prevalence, and human mobility data is needed to attribute causal weights to these drivers.

In summary, Bangladesh has entered a sustained hyperendemic dengue era (ongoing as of October 2025). Public health planning, resource allocation, and intervention strategies must be fundamentally adapted to this new reality to mitigate morbidity, mortality, and healthcare system strain.

## Conclusion

In conclusion, the trajectory of dengue in Bangladesh has been fundamentally reshaped by a series of structural breaks, culminating in a sustained hyperendemic regime that has persisted from 2019 through October 2025. The consistent August peak (seasonal ratio ≈ 180) represents an enormous annual challenge to the healthcare system, superimposed on this new high-transmission baseline. The confirmation that hyperendemic transmission has been sustained for so many months demonstrates this is not a temporary crisis but an established new reality. The recent break in 2024 within this regime is a stark warning that the situation may still be escalating. Recognizing these discrete epidemiological regimes is not merely an academic exercise but a crucial prerequisite for developing adaptive, effective, and realistic public health responses. The era of responding to dengue based on past, lower-intensity regimes is over; preparedness and policy must be scaled to the new reality of a persistent, high-magnitude hyperendemic state that has been unequivocally established through 2025.

## Supporting information

S1 File Break points in dengue admission timeline. (CSV)

S2 File Cluster evaluation with Silhouette Scores. (CSV)

S3 File Epidemiological metrics. (CSV)

S4 File Determined Markov Regimes (Statistics). (CSV)

S5 File Comparison of methods for consensus count in break point detection. (CSV)

S6 File Determined regimes from K means clustering (statistics). (CSV)

S7 File A total comprehensive epidemiological analysis report. (DOCX)

S8 File Flowchart of the study. (TIF)

S9 File Database. (XLSX)

## Abbreviations

DGHS: Directorate General of Health Services
IEDCR: Institute of Epidemiology Disease Control and Research
STL: Seasonal-Trend decomposition using LOESS
LOESS: Locally Estimated Scatterplot Smoothing
ARIMA: Autoregressive Integrated Moving Average
SARIMA: Seasonal Autoregressive Integrated Moving Average
