# Supplementary material for: The emergence of hyperendemic dengue in Bangladesh: An ecological study of structural breaks and transmission regime shifts, 2008–2025
Source: PLoS One. 2026 Feb 20;21(2):e0343246. doi: 10.1371/journal.pone.0343246 (PMC12923051; doi:10.1371/journal.pone.0343246)
Supplement: S7 File — (DOCX) [file pone.0343246.s007.docx]

Comprehensive Epidemiological Structural Break Analysis: Dengue in Bangladesh

Detailed investigation of transmission regime shifts and epidemiological phases

# Epidemiological Analysis: Dengue Admitted

## Data Overview

Analysis period: 2008-01 to 2025-10

Total months: 214

Total cases: 711,618

Mean monthly cases: 3325.3

Data quality notes: Good quality

## Epidemiological Metrics

Basic Statistics:

Coefficient of Variation: 3.226

Maximum monthly cases: 73,023

Outbreak Characteristics:

Major outbreaks (>3σ): 0

Outbreak frequency: 4.7%

Trend Analysis:

Estimated annual growth: 23.1%

Seasonality:

Peak month: 8

Seasonal ratio: 180.1

Calculation Methods:

outbreak_detection: Z-scores relative to 12-month moving average

trend_analysis: Linear regression on time index

seasonality: Monthly averages across complete series

## Identified Structural Breaks

Total breaks detected: 10

Breakpoint Detection Method Summary:

PELT algorithm: 1 breaks

Binary Segmentation: 10 breaks

Window-based method: 7 breaks

Average consensus score: 1.50/3

Break 1: 2021-05 (Consensus: 3/3, Methods: PELT, BinarySeg, Window, Mean ratio: 13.12x)

Break 2: 2020-02 (Consensus: 2/3, Methods: BinarySeg, Window, Mean ratio: 9.42x)

Break 3: 2015-07 (Consensus: 2/3, Methods: BinarySeg, Window, Mean ratio: 82.55x)

Break 4: 2011-05 (Consensus: 2/3, Methods: BinarySeg, Window, Mean ratio: 80.28x)

Break 5: 2023-06 (Consensus: 1/3, Methods: BinarySeg, Mean ratio: 14.01x)

Break 6: 2024-09 (Consensus: 1/3, Methods: BinarySeg, Mean ratio: 4.26x)

Break 7: 2018-06 (Consensus: 1/3, Methods: BinarySeg, Mean ratio: 53.27x)

Break 8: 2018-01 (Consensus: 1/3, Methods: Window, Mean ratio: 48.78x)

Break 9: 2023-11 (Consensus: 1/3, Methods: Window, Mean ratio: 3.69x)

Break 10: 2017-03 (Consensus: 1/3, Methods: BinarySeg, Mean ratio: 47.41x)

## Transmission Regimes

Optimal number of regimes: 3

Silhouette score: 0.867

Cluster Evaluation Scores:

2 clusters: silhouette score = 0.854

3 clusters: silhouette score = 0.867

4 clusters: silhouette score = 0.865

5 clusters: silhouette score = 0.834

Regime 1: 2008-01 to 2022-05 (mean=362, CV=2.95, Outbreaks: 5.0%)

Regime 2: 2023-04 to 2024-03 (mean=26289, CV=1.18, Outbreaks: 0.0%)

Regime 3: 2019-03 to 2025-05 (mean=7416, CV=1.57, Outbreaks: 5.6%)

## Seasonality Analysis

Seasonality strength: 0.335

Mean amplitude: 9355.64

Decomposition method: STL with period=12

Seasonal pattern breaks detected: 1

## Markov Regime Switching Analysis

Number of regimes: 3

AIC: 3252.3

BIC: 3292.7

Model Specification:

Model type: MarkovRegression

Trend specification: constant

Switching variance: True

Convergence status: failed

Regime 1: 2008-01 to 2023-04 (mean=47, std=61)

Regime 2: 2008-08 to 2025-06 (mean=1288, std=1407)

Regime 3: 2019-07 to 2025-10 (mean=26127, std=21132)

## Statistical Tests

Zivot-Andrews Test (Unit Root with Structural Break):

Test statistic: -8.277

p-value: 0.000

Structural break detected: YES

Test parameters: maxlag=12

Stationary series (ADF): YES

ADF test parameters: Default ADF test with automatic lag selection

Stationary series (PP): YES

PP test parameters: Default PP test with automatic lag selection


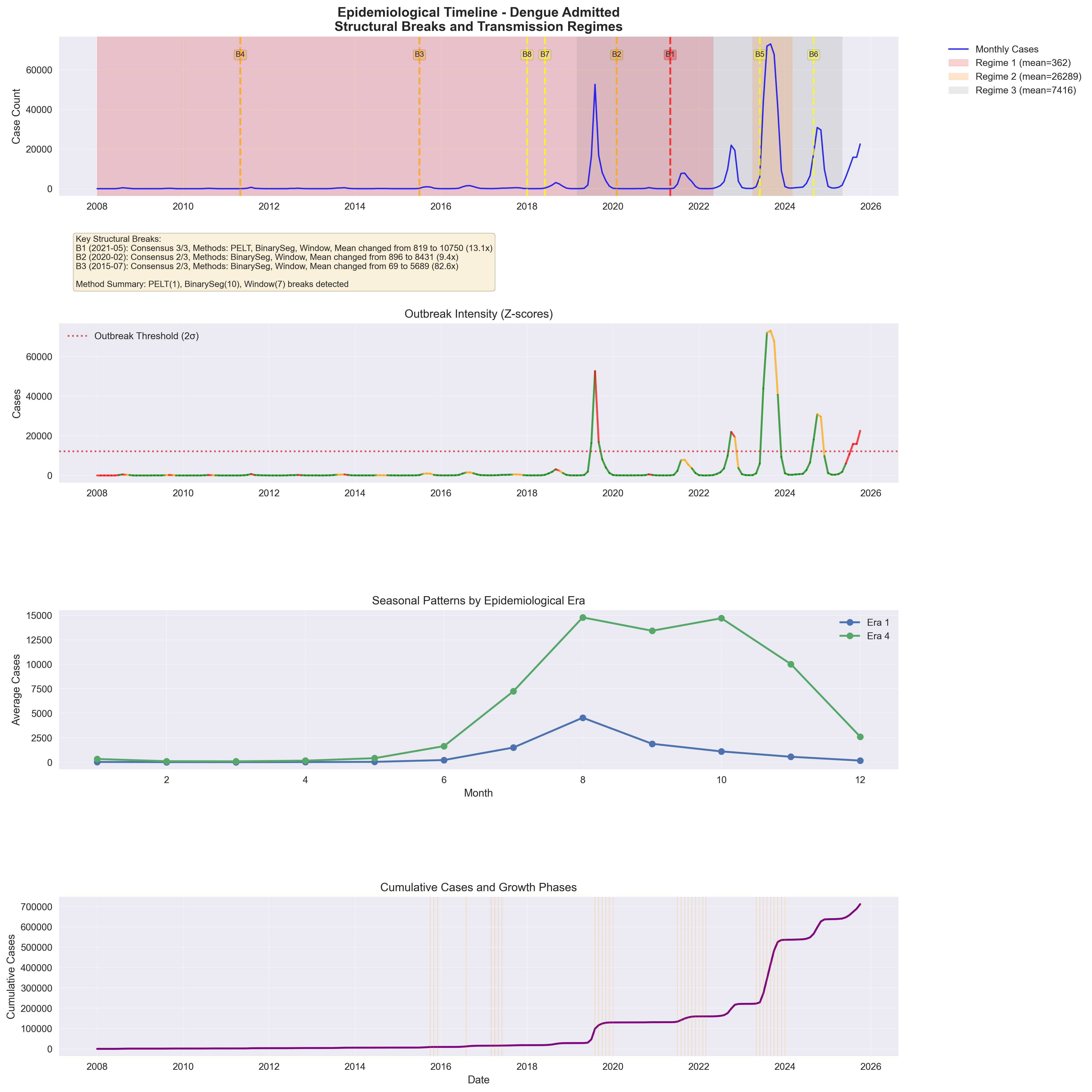


Figure: Dengue_Admitted_epidemiological_timeline.png


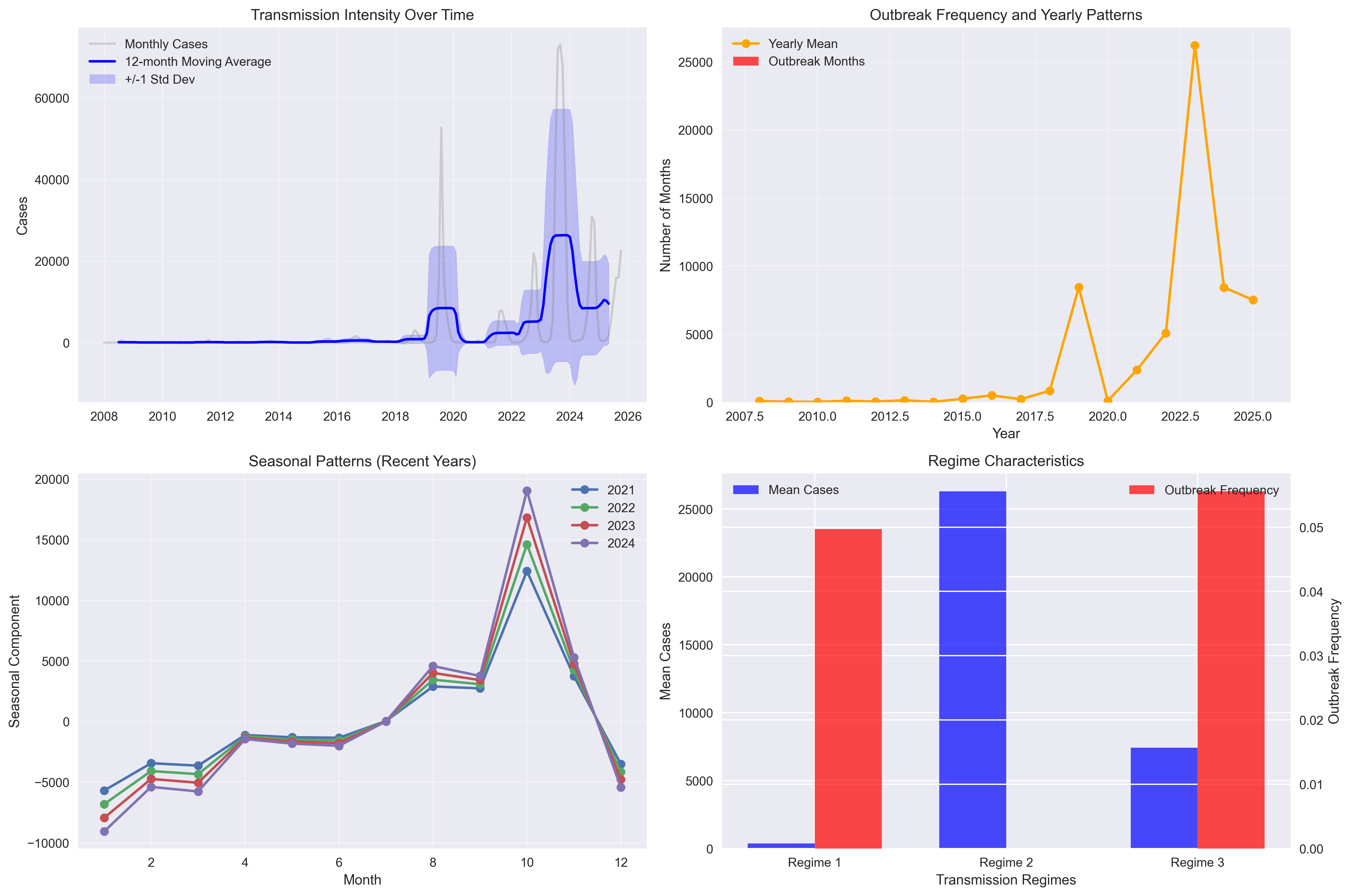


Figure: Dengue_Admitted_epidemiological_metrics.png


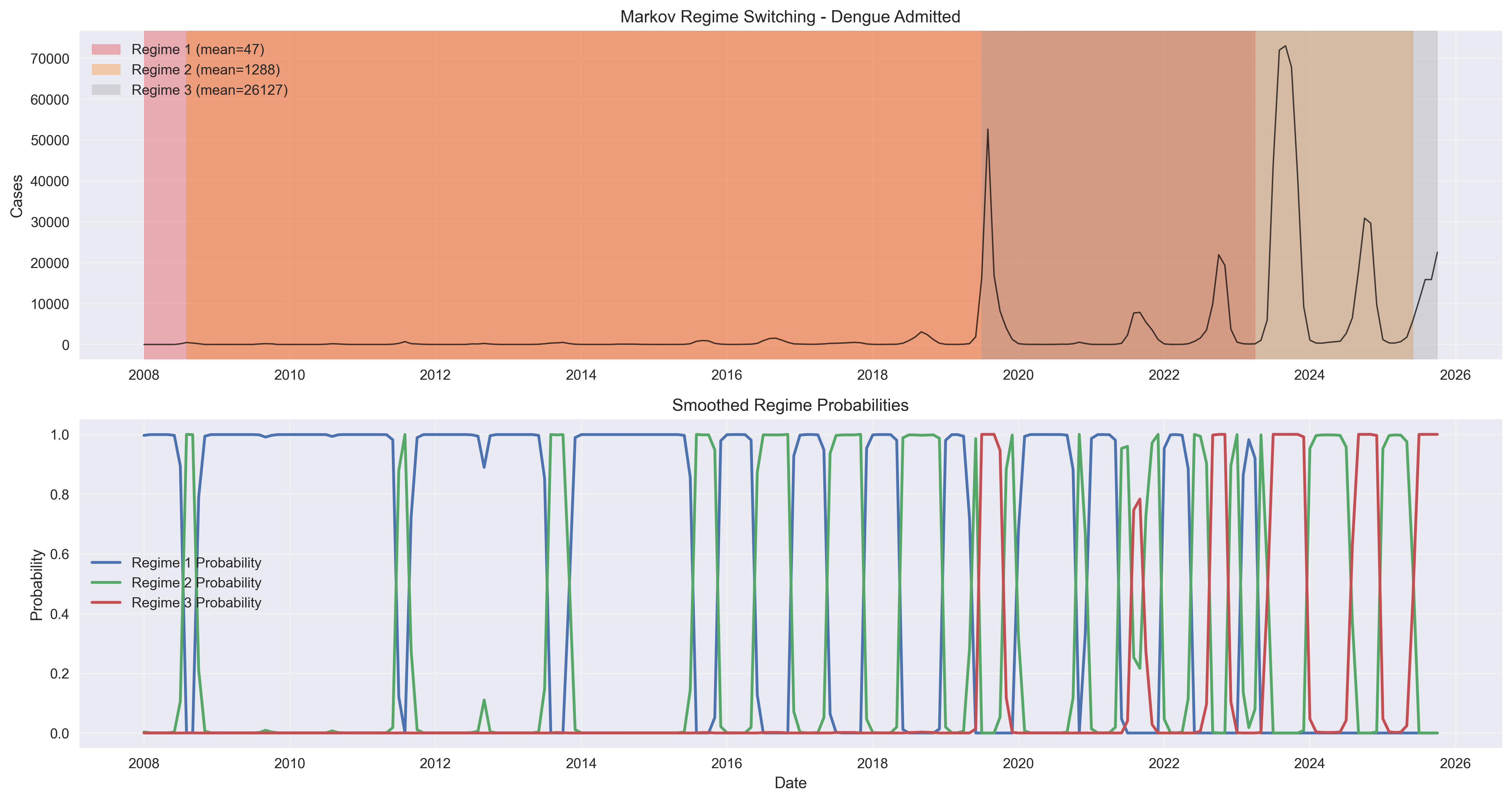


Figure: Dengue_Admitted_markov_regimes.png

Dengue Admitted Break Points: A:/Library/Articles and Research by Pratyay Hasan/Original Articles/Dengue Evolution Epidemiology\Comprehensive_Epi_Breaks_Results\Tables\Dengue_Admitted_break_points.csv

Dengue Admitted Method Comparison: A:/Library/Articles and Research by Pratyay Hasan/Original Articles/Dengue Evolution Epidemiology\Comprehensive_Epi_Breaks_Results\Tables\Dengue_Admitted_method_comparison.csv

Dengue Admitted Regime Statistics: A:/Library/Articles and Research by Pratyay Hasan/Original Articles/Dengue Evolution Epidemiology\Comprehensive_Epi_Breaks_Results\Tables\Dengue_Admitted_regime_statistics.csv

Dengue Admitted Cluster Evaluation: A:/Library/Articles and Research by Pratyay Hasan/Original Articles/Dengue Evolution Epidemiology\Comprehensive_Epi_Breaks_Results\Tables\Dengue_Admitted_cluster_evaluation.csv

Dengue Admitted Epidemiological Metrics: A:/Library/Articles and Research by Pratyay Hasan/Original Articles/Dengue Evolution Epidemiology\Comprehensive_Epi_Breaks_Results\Tables\Dengue_Admitted_epidemiological_metrics.csv

Dengue Admitted Markov Regimes: A:/Library/Articles and Research by Pratyay Hasan/Original Articles/Dengue Evolution Epidemiology\Comprehensive_Epi_Breaks_Results\Tables\Dengue_Admitted_markov_regimes.csv
